# Development and validation of the Oxford Benchmark Scale for Rating Vaccine Technologies (OBSRVT), a scale for assessing public attitudes to next-generation vaccine delivery technologies

**DOI:** 10.1080/21645515.2025.2469994

**Published:** 2025-03-03

**Authors:** Jonathan Kantor, Robert C. Carlisle, Samantha Vanderslott, Andrew J. Pollard, Michael Morrison

**Affiliations:** a Oxford Vaccine Group, University of Oxford, Oxford, UK; b Biomedical Ultrasonics, Biotherapy, and Biopharmaceuticals Laboratory (BUBBL), Institute of Biomedical Engineering, Department of Engineering Science, University of Oxford, Oxford, UK; c Center for Clinical Epidemiology and Biostatistics, University of Pennsylvania Perelman School of Medicine, Philadelphia, PA, USA; d Centre for Health, Law, and Emerging Technologies (HeLEX), University of Oxford, Oxford, UK

**Keywords:** Vaccine delivery technology, vaccines, de-risking, public health, validation study, behavior rating scale, pain

## Abstract

Next-generation vaccine delivery technologies may provide significant gains from both a technical and behavioral standpoint, but no scale has yet been developed to assess public attitudes to novel vaccine delivery technologies. We therefore performed a cross-sectional validation study that included 1,001 demographically representative participants from the UK and US to develop and validate a novel scale, the Oxford Benchmark Scale for Rating Vaccine Technologies (OBSRVT). A sample of 500 UK participants was used to perform exploratory factor analysis with categorical variables (using a polychoric correlation matrix) followed by promax oblique factor rotation to develop the initial model. This yielded a 15-item 4-domain scale with domains including acceptance (6 items), effectiveness (4 items), comfort (3 items), and convenience (2 items). This model was tested for robustness on a 501-participant demographically representative sample from the US. A confirmatory factor analysis with a Satorra-Bentler scaled test statistic was performed, which demonstrated adequate goodness of fit statistics including the root mean squared error of approximation (0.057), standardized root mean squared residual (0.053), and comparative fit index (0.938). Reliability as internal consistency was excellent (alpha = 0.92). Convergent validity with the Oxford Needle Experience Scale was supported by an adequate correlation (*r* = 0.31, *p* < .0001), while discriminant validity was supported by a lack of correlation with an unrelated question (*r* = -0.03, *p* < .0001). These findings suggest that the OBSRVT scale represents a feasible, valid, and reliable scale that could be used to gauge the acceptability of existing and future vaccine delivery technologies, and further investigation and testing should be considered.

## Introduction

Developing novel vaccine delivery technologies is an international public health priority, driven by both the ongoing challenge of vaccine hesitancy and the need for more efficient, deployable, and innovative approaches to vaccine delivery.^1,2^ While traditional needle and syringe-based vaccination has represented the standard of care for decades, next-generation vaccine delivery technologies such as nasal sprays, microneedle patches, oral vaccines, and ultrasound are increasingly being investigated as alternative, and potentially superior, approaches to vaccine delivery.^3–7^

Understanding public willingness to consider the uptake of next-generation vaccine delivery technologies is important for two central reasons. First, alternative vaccine delivery technologies may help overcome some of the technical and logistical barriers to vaccination – cold storage, enhancing immune response, risk of sharps injuries, and cost – that could lead to more seamless deployment.^6,7^ Second, these approaches may help mitigate some of the hesitancy surrounding vaccination, a phenomenon that may present an even greater, or at least more intransigent, barrier to effective vaccine delivery than deployment and efficacy challenges.^8^

Yet despite decades of work in developing alternatives to needle and syringe-based vaccination, public attitudes to these individual approaches remains unknown, and little has been done to develop a validated approach to assessing patient and public preferences for novel vaccine delivery technologies.^9^ This is a critical lacuna, as our previous work has suggested that a fear of needles may be the only modifiable risk factor for vaccine hesitancy.^10^ That said, novel technologies deployed in a medical setting are also sometimes themselves associated with increased mistrust.^11–13^ Therefore, while developing needle-free approaches to vaccine delivery may represent a powerful modulatory tool in the armamentarium of public health researchers to mitigate vaccine hesitancy, it remains difficult to determine the net value of novel delivery technologies from the public perspective.

As new vaccine delivery approaches are considered, developed, and trialed, it will be crucial to provide researchers with validated instruments so that the preferences of the public and trial participants can be studied, considered, and addressed. On a practical level, while funding agencies and pharmaceutical companies are highly invested in developing new delivery approaches, a validated scale to assess acceptance and attitudes to specific approaches of interest could help with de-risking these technologies at an early stage, helping to target investment and research across the timeline of technological development.^14^

We therefore sought to develop and validate a feasible, psychometrically sound, and multidimensional benchmark scale – the Oxford Benchmark Scale for Rating Vaccine Technologies (OBSRVT) – that could be used at all phases of alternative vaccine delivery approach development to gauge the relative preferences of the public for these technologies. Ultimately, such a scale may provide evidence of the putative benefits of novel, and sometimes costly, delivery technologies from a societal standpoint that could help tailor future development to better align with public preferences while also helping to justify the research and development of these technologies. This scale will hopefully lead to a better understanding of the barriers to vaccine program delivery in all its myriad forms, ultimately resulting in enhanced vaccine coverage and improved public health outcomes.

## Materials and methods

### Data collection, population, and overall approach

Our overall methodology was similar to our prior work in developing instruments to assess needle attitudes and vaccine hesitancy.^15–18^ Initially, we developed a series of questionnaire-based surveys in Qualtrics (Qualtrics International, Provo, Utah, USA) with responses solicited using a survey panel design through Prolific Academic (Oxford, UK) from two separate 50-member cohorts. The first cohort was a convenience sample and the second represented a vaccine hesitant group who self-identified as never having received a vaccination for COVID-19.

After this initial exploratory study, two separate 500-respondent demographically representative samples from the UK and USA (based on UK Office of National Statistics or 2015 US national census bureau data, respectively, and matched by stratification on age, sex, and ethnicity) were targeted to develop the OBSRVT scale. The UK data were used for the exploratory factor analysis (EFA), while US data were used for confirmatory factor analysis (CFA) and reliability and validity testing. The US sample served to assess the scale’s robustness across a distinct, but similar, population, consistent with prior methodological approaches in this area. Sample sizes of approximately 500 participants in each cohort were selected based on two considerations. First, best practices suggest including approximately 10 participants per item in EFA and CFA^19^; with a total of 40 candidate items for inclusion, we conservatively estimated 500 participants per cohort. Second, our aim was to use demographically representative samples to improve our generalizability, and including a 500-subject sample size in each population permitted representative demographic stratification.

To maximize the generalizability of our scale to all current and future vaccine delivery technologies, we chose to benchmark all responses against respondents’ feelings about vaccine delivery with a traditional needle and syringe. Responses to all items in the scale were therefore scored on a 5-point Likert scale ranging from ‘much less than with a needle and syringe’ to ‘much more than with a needle and syringe,’ with a neutral option ‘the same as with a needle and syringe’ included. Items were reverse coded, as needed, to yield a final scale in which higher scores represent a greater degree of support for the delivery technology under question than with a needle and syringe.

This research was approved by the University of Oxford Medical Sciences Interdivisional Research Ethics Committee (approval reference R81585/RE001). Participants were reimbursed less than £5 for their time. All participants provided informed consent for participation and were permitted to withdraw at any time. Demographic details were self-declared and linked to responses using a unique 25-character alphanumeric identifier. Demographic data included age, self-identified gender, self-identified race and ethnicity, employment status, educational background, household income, and COVID-19 vaccination status.

Qualitative analyses were performed using NVivo release 1.4 (QSR International, Burlington, Massachusetts, USA) and statistical analyses were performed using Stata for Mac version 16 (Stata Corporation, College Station, Texas, USA). A p-value of 0.05 was considered significant for all analyses.

### Domain and item generation

To develop a set of domains for consideration, we relied on a 3-part approach. First, a literature review was performed to determine what domains should be considered based on historical precedent. Second, the two 50-member survey cohorts were used to expand the range of domains for inclusion by asking both open- and closed-answer questions regarding priorities for vaccine delivery technologies. Finally, this qualitative research was followed by iterative feedback from the Oxford Vaccine Group and the Engineering Science biology/in vivo cluster meetings.

Closed-ended responses were reported with a 5-point Likert scale while open-ended responses were recorded in free text format. Open-ended questions included those such as: ‘What are your priorities when deciding on how much you value a vaccine delivery technology?’ A draft survey was also provided, with respondents given the opportunity to answer whether any items should be added, modified, or deleted, and whether the answer choices and questions were clear and appropriate.^15,16,19,20^

### Domain and item reduction and exploratory factor analysis

Item reduction was performed by reassessing domain relevance, deleting redundant items, examining polychoric (item-item) and polyserial (item-test) correlations, and deleting items with low communality or factor loadings.^21^ Both Bartlett’s test of sphericity and the Kaiser-Meyer-Olkin (KMO) measure of sampling adequacy were assessed to confirm that data were amenable to factor analysis.^22^ A combination of an iterative approach, parallel analysis, scree plot, and assessment of the minimum average partial correlation were used to assess the number of domains to retain for inclusion.^20,23,24^ EFA with categorical variables was performed using a polychoric correlation matrix with iterated principal factor analysis followed by promax oblique factor rotation.^25,26^

### Confirmatory factor analysis

We performed confirmatory maximum likelihood factor analysis with structural equation modeling and a Satorra-Bentler scaled test statistic on the US sample using the model developed in the UK sample to confirm the robustness of our model. Goodness of fit indices were assessed, including the root mean squared error of approximation (RMSEA), the standardized root mean squared residual (SRMR), and the comparative fit index (CFI). Values of RMSEA ≤ 0.08, SRMR ≤ 0.08, and CFI ≥ 0.90 suggest acceptable fit, while values of RMSEA ≤ 0.05, SRMR ≤ 0.06, and CFI ≥ 0.95 suggest excellent fit.^27,28^

### Reliability and validity

Reliability as internal consistency was assessed using Cronbach’s alpha for the overall OBSRVT scale and each subscale.^29^

We assessed convergent validity using the Oxford Needle Experience (ONE) scale, a validated scale that measures public attitudes to needles.^15^ Discriminant validity was assessed using a single question regarding political outlook (‘I consider myself to be a conservative person’). For both convergent and discriminant validity, the Pearson correlation coefficient was used.

## Results

### Characteristics of the representative samples

#### UK EFA sample

A total of 541 individuals initially accessed our survey; of these, 21 failed to complete the survey (yielding a completion rate of 96.1%) and 20 (3.8%) failed at least one attention check, yielding 500 unique participants for analysis. The median (interquartile range [IQR]) age was 48 (33, 59) and respondents ranged in age from 18 to 82. Overall, 52.3% of respondents identified as female and the median (IQR) household income was £45,000 (26,000, 65000). Additionally, 15.6% of the participants identified as nonwhite, 42.3% reported having no children, 47.3% reported working full-time, and 91.4% reported receiving at least one vaccination for COVID-19.

#### US CFA sample

A total of 527 individuals initially accessed our survey; of these, 24 failed to complete the survey (yielding a completion rate of 95.4%) and 22 (4.4%) failed at least one attention check, yielding 501 unique participants for analysis. The median (interquartile range [IQR]) age was 47 (32, 59) and respondents ranged in age from 18 to 95. Overall, 50% of respondents identified as female and the median (IQR) household income was $65,000 (40,000, 100,000). Additionally, 34.3% of the participants identified as nonwhite, 44.5% reported having no children, 56.7% reported working full-time, and 81.4% reported receiving at least one vaccination for COVID-19.

### Domain and item reduction and exploratory factor analysis

Our iterative approach yielded 10 potential domains including effectiveness, safety (including risks and side effects), ease of use (convenience), acceptance, comfort, cultural acceptability, likelihood of recommendation to others, perceived advantages, perceived disadvantages, and concerns (including anxiety or fear) associated with the delivery approach. We explored a total of 40 items for inclusion in the scale; 25 items were ultimately culled due to redundancy, low item correlations, and low factor loadings, yielding a final 15-item scale.

Bartlett’s test of sphericity (*p* < .0001) suggested the data were appropriate for EFA, which was confirmed by the KMO measure of sampling adequacy, which was excellent at 0.95. A scree plot, minimum average partial correlation analysis, and parallel analysis suggested a 2 to 4 factor solution.^23,30,31^

### Domains included in the final scale

The four domains ultimately included in the scale were acceptance (6 items), effectiveness (4 items), confidence (3 items), and concerns (2 items). The acceptance domain reflects a generally positive response to the vaccine delivery technology in question, including likelihood of vaccination, willingness, preference, and comfort. Effectiveness, in contrast, reflects the degree to which the respondent believes that the delivery technology will be effective in vaccine delivery, providing immunity, and protection. Confidence highlights the perceived ease of use, ability of health care professionals to deliver the vaccine, and perception of vaccine safety. Finally, the concerns domain addresses fear or worry regarding side effects or safety associated with vaccine delivery using the technology under study.

Factor loadings are included in Table 1 and the OBSRVT scale is presented in Table 2.

**Table 1.** Rotated factor loadings for the OBSRVT. Factor loadings >0.40 (our a priori minimum for consideration) are bolded.

Item	Acceptance	Effectiveness	Confidence	Concerns
I would be likely to get vaccinated if this vaccine delivery method were available.	**0.9377**	−0.0221	−0.0817	−0.0374
I am willing to try this vaccine delivery method in the future.	**0.8894**	−0.0169	0.0593	−0.0194
I would prefer this vaccine delivery method.	**0.8703**	−0.0124	0.0634	0.0175
I believe this vaccine delivery method should be widely adopted.	**0.7570**	0.2215	−0.0222	−0.0429
I would recommend this vaccine delivery method to friends and family.	**0.7560**	0.1965	0.0220	−0.0286
This vaccine delivery method is comfortable.	**0.6434**	−0.2154	0.0945	0.0015
I trust that this delivery method will provide immunity.	0.0166	**0.9844**	−0.0676	−0.0112
I am confident in the long-term effectiveness of this vaccine delivery method.	0.0428	**0.9013**	−0.0279	0.0213
I believe this vaccine delivery method would be effective in preventing disease.	−0.0230	**0.8884**	0.0284	−0.0126
I believe this method would be effective in delivering the vaccine as needed.	0.0532	**0.7948**	0.1292	0.0155
This vaccine delivery method can be easily used for the general population.	0.1562	−0.0909	**0.7464**	0.0712
I am confident that health professionals can safely deliver vaccines using this method.	0.0431	0.1287	**0.7301**	0.0389
I feel this vaccine delivery method is safe.	0.0623	0.0129	**0.4538**	−0.2355
I am fearful of the potential side effects of this vaccine delivery method.	−0.0516	0.0352	0.0740	**0.8937**
I worry about the safety of this vaccine delivery method.	−0.0497	−0.0307	−0.0155	**0.8479**

**Table 2.** The OBSRVT scale. All items are scored using a 5-point Likert scale ranging from much less than with a needle and syringe to much more than with a needle and syringe. Higher scores thus reflect a greater degree of desirability relative to traditional needle and syringe-based vaccination, with a score of 45 suggesting equivalence to needle and syringe-based vaccination. Items 14 and 15 are reverse scored. The total score can range from 15 to 75.

#	Item	Domain
1	I would be likely to get vaccinated if this vaccine delivery method were available.	Acceptance
2	I am willing to try this vaccine delivery method in the future.	Acceptance
3	I would prefer this vaccine delivery method.	Acceptance
4	I believe this vaccine delivery method should be widely adopted.	Acceptance
5	I would recommend this vaccine delivery method to friends and family.	Acceptance
6	This vaccine delivery method is comfortable.	Acceptance
7	I trust that this delivery method will provide immunity.	Effectiveness
8	I am confident in the long-term effectiveness of this vaccine delivery method.	Effectiveness
9	I believe this vaccine delivery method would be effective in preventing disease.	Effectiveness
10	I believe this method would be effective in delivering the vaccine as needed.	Effectiveness
11	This vaccine delivery method can be easily used for the general population.	Confidence
12	I am confident that health professionals can safely deliver vaccines using this method.	Confidence
13	I feel this vaccine delivery method is safe.	Confidence
14	I am fearful of the potential side effects of this vaccine delivery method.	Concerns
15	I worry about the safety of this vaccine delivery method.	Concerns

### Confirmatory factor analysis

Using the final 4-factor 15-item model and structural equation modeling with a Satorra-Bentler scaled test statistic on the US sample, we demonstrated very good goodness of fit characteristics, with RMSEA = 0.057, SRMR = 0.053, and CFI = 0.958.

### Scale reliability and validity

Reliability as internal consistency was measured using Cronbach’s alpha; values for the overall scale were very good at 0.92. Values for each of the four subscales are included in Table 3.

**Table 3.** Subscale and overall reliability (assessed using Cronbach’s alpha).

	UK Sample	US Sample
Acceptance	0.91	0.92
Effectiveness	0.92	0.93
Confidence	0.70	0.71
Concerns	0.84	0.81
Overall OBSRVT	**0.91**	**0.92**

Convergent validity between the OBSRVT score and the Oxford Needle Experience (ONE) scale score was 0.31 (*p* < .0001), and discriminant validity between the OBSRVT scale and a single unrelated question (‘I consider myself to be a conservative person’) was −0.03 (*p* < .0001), supporting our validity argument.

## Discussion

This manuscript outlines the initial development and validation of the OBSRVT, the first scale to assess the public’s attitude to novel vaccine delivery technologies. We developed a concise, feasible 15-item scale over four domains: acceptance, effectiveness, confidence, and concerns. By benchmarking responses relative to needle and syringe-based vaccination, we aimed to maximize the utility of the scale in assessing both existing and future vaccine delivery technologies. This may represent an important next step in better understanding public priorities regarding novel vaccine delivery technologies.

Indeed, prior studies evaluating a range of delivery technologies from microneedle array patch approaches to nasal sprays routinely report outcomes such as efficacy, safety, and stability,^32–35^ but acceptance is generally reported without a validated or standardized instrument.^36,37^ Given the importance of public buy-in, both to decrease hesitancy and justify the significant public and private cost of this research, this gap is striking and highlights the potential value of the present work.

We adopted a benchmarking approach to rate novel vaccine delivery technologies relative to the ubiquitous needle and syringe-based vaccination approach. Benchmarking not only allows us to assess preferences relative to needle and syringe vaccines, but also to compare different alternative vaccine delivery technologies to each other, since each is rated relative to a stable, familiar, and immutable benchmark.

Each of the four domains in our scale – acceptance, effectiveness, confidence, and concerns – includes a different number of items. Since the scale is scored on a Likert scale ranging from 1 (‘much less than with a needle and syringe’) to 5 (‘much more than with a needle and syringe’), a score of 3 on each item suggests equivalence to needle and syringe (‘the same as with a needle and syringe’). Thus, the neutral cutoffs for each subscale, as outlined in Table 2, are 18, 12, 9, and 6 (yielding an overall scale cutoff of 45), which allows for easy benchmarking of the technology under investigation; values higher than the cutoffs suggest that the technology under investigation is preferred to a needle and syringe. This approach is particularly powerful in the dynamic environment of vaccine delivery technology development, as the OBSRVT is flexible and adaptable enough to be used to evaluate future technologies that have not yet been considered.

The benchmarking approach is also powerful on a practical level; sample data (Table 4) for an assessment of a theoretical nasal spray vaccine, for example, suggest that the overall feelings regarding this approach in the UK and US are similar, with the same median score in each population, bolstering our validity argument. Even these sample data could suggest to researchers, funders, and governments that the public largely sees nasal spray as superior to needle and syringe-based vaccination, but that concerns regarding effectiveness (with scores slightly below the threshold, suggesting concern relative to needle and syringe-based vaccination) would need to be addressed carefully to encourage broad adoption. These findings also further support the face validity of the OBSRVT scale, since (as expected) participants were enthusiastic about the potential for nasal vaccine delivery based on acceptance scores while – not surprisingly – they had some residual concern regarding effectiveness, presumably given the relative novelty of the technology.

**Table 4.** Representative comparison of scores for the OBSRVT scale when assessing a nasal spray vaccine between UK and US samples. The table presents the median and interquartile range (IQR) values for responses in the domains of acceptance, effectiveness, confidence, and concerns, as well as the overall OBSRVT score. P-values are derived from the Mann-Whitney U test, assessing the statistical significance of differences in the distributions between the two independent samples. Cutoffs for needle and syringe based equivalence are based on a score of 3 for each Likert scale item; this is computed easily based on the number of items in each domain. A p-value less than 0.05 suggests a statistically significant difference.

	UK Sample Median (IQR)	US Sample Median (IQR)	Cutoffs for needle and syringe equivalence	p-value
Acceptance	21 (18–25)	21 (17–24)	18	0.02
Effectiveness	10 (8–12)	11 (8–12)	12	0.07
Confidence	10 (9–12)	10 (9–11)	9	0.02
Concerns	6 (6–7)	6 (4–7)	6	0.27
OBSRVT Overall	48 (43–54)	48 (41–54)	45	0.17

Our work has several other strengths. First, we used a rigorous iterative process for domain and item development, including qualitative data from open-ended questions, to ensure that we could capture public priorities adequately. Second, we specifically included a vaccine hesitant cohort when initially soliciting feedback to increase the chances that we would capture priorities of a traditionally marginalized – yet critical for increasing novel vaccine adoption – group. Third, we used large demographically representative samples for our exploratory and confirmatory factor analyses to improve the generalizability of our findings. Fourth, to assess the robustness of our model, we used a UK sample for exploratory factor analysis and a US sample for confirmatory factor analysis. Fifth, we performed our EFA using polychoric correlation matrices, an approach designed specifically for categorical data, to ensure that the factor analysis was performed appropriately given the Likert-scale responses. Finally, as alluded to above, the face validity of the scale was supported by the similarity in the baseline scores between the UK and US populations as well as the lower scores seen for the effectiveness subscale.

By design, there is some overlap between the items included within each of the scale’s domains. For example, in the Confidence domain, item 12 (“I am confident that health professionals can safely deliver vaccines using this method”) addresses confidence in the perceived ability of health professionals to *practically use* the delivery technology in question, while item 13 (“I feel this vaccine delivery method is safe”) addresses a more general sense of confidence in the *overall perceived safety* of the technology itself. The distinction between these items may be particularly notable when exploring novel vaccine delivery technologies that may be perceived as technically difficult to deliver in a reproducible and consistent manner.

Limitations of our study include our use of a survey panel for subject recruitment, so that participants may not truly reflect the population of interest, though this was mitigated by relying on large demographically representative samples. Indeed, the essentially identical scores seen in the UK and US populations (Table 4) suggests that our findings are indeed robust and thus likely representative. Second, our domains include an uneven number of items, and only two items were included in the concerns domain; still, since those items address the two key area of potential concern for participants – side effects and safety – and since their loading and structure is statistically valid, the practical utility of the factorial structure of our scale is supported. Third, while we included representative samples from both the UK and US in this study, future studies exploring whether our scale is valid across disparate cultural contexts, as well as invariance testing and test-retest reliability assessments, may be warranted. Fourth, most of the items in our scale were framed to elicit acceptance; while this is in part a function of our domain choices, and we included two items with reverse scoring in the Concerns domain, it is possible that this approach could introduce bias. Finally, given the potential for baseline attitudes to needle and syringe-based vaccination to change over time, individual ratings may not remain stable. Thus, future studies assessing the responsiveness of the OBSRVT and its subscales – that is, how individual scores change over time as a result of the evolution of preferences, changes in circumstance, or a history of exposure – may be considered.

This manuscript presents data on the development and validation of the OBSRVT, the first scale designed specifically to assess the public’s attitudes to next-generation vaccine delivery technologies. Given the public and private outlay of research funding in these areas,^38,39^ and the potential for such technologies to fundamentally shift the ways in which vaccine delivery is effectuated, a formal validated scale is necessary to better assess public buy-in, better justify research funding, better aid in de-risking technologies, and better understand potential acceptance bottlenecks prior to full vaccine technology deployment. By developing a short yet comprehensive 15-item, 4-domain scale that captures key considerations for vaccine delivery technologies – acceptance, effectiveness, confidence, and concerns – we hope that our work may serve as a tool for investigators as vaccine delivery technologies continue to evolve.

## Data statement

Data are available from the corresponding author.
